# Dual-energy CT pulmonary angiography using dual-flow mixture bolus tracking: potential for contrast medium and radiation exposure reduction

**DOI:** 10.1186/s13244-026-02329-x

**Published:** 2026-06-20

**Authors:** Mingjue Jian, Lisheng Huang, Chenxi Zhang, Xiang Qin, Haixia Li, Jiafang Wu, Mingwang Chen, Li Ding, Xiaomei Li, Yikai Xu, Zhao Chen, Chenggong Yan

**Affiliations:** 1 Department of Medical Imaging Center, Nanfang Hospital, Southern Medical University, Guangzhou, China; 2 Bayer Healthcare Company Limited, Radiology, Guangzhou, China; 3 School of Laboratory Medicine and Biotechnology, Southern Medical University, Guangzhou, China

**Keywords:** CT pulmonary angiography, Dual-energy CT, Pulmonary embolism, Radiation dose, Virtual monoenergetic imaging

## Abstract

**Objectives:**

To assess the feasibility and image quality of a bolus tracking protocol with dual-flow mixture for contrast medium volume and radiation dose reduction in dual-energy CT pulmonary angiography (CTPA).

**Materials and methods:**

Patients with suspected pulmonary embolism (PE) were prospectively included and randomly divided into the dual low-dose or routine CTPA groups. Virtual monoenergetic imaging results at 40 keV for the dual low-dose CTPA group were compared to 100-kV polyenergetic CT images from the routine CTPA group. Attenuation, noise, contrast-to-noise ratio, and figure of merit were determined in multiple pulmonary arteries. Qualitative image quality and PE detection were independently rated by two radiologists.

**Results:**

The dual low-dose CTPA group showed a significantly higher attenuation in 40-keV virtual monoenergetic imaging (1027.0 ± 287.6 HU vs 391.8 ± 109.0 HU; *p* < 0.001) with a comparable median contrast-to-noise ratio (33.6 vs 41.0; *p* = 0.115) and superior figure of merit (711.4 vs 461.5; *p* = 0.006) in the pulmonary trunk in relation to that observed in the routine CTPA group. Superior vena cava artifacts were reduced (both *p* ≤ 0.002), while pulmonary branch visualization was preserved (*p* = 0.660 and 0.763). Effective radiation dose was lower in the dual low-dose CTPA group (1.61 mSv vs 3.62 mSv; *p* < 0.001), while PE diagnostic performance did not differ significantly between the two protocols.

**Conclusion:**

The dual-flow mixture bolus-tracking protocol showed technical feasibility for dual-energy CTPA, achieving substantial reductions in radiation exposure and contrast medium volume while maintaining diagnostically acceptable image quality.

**Critical relevance statement:**

The dual-flow mixture bolus-tracking protocol with dual-energy CT maintained feasible image quality for PE assessment, facilitating contrast medium volume and radiation exposure reduction.

**Trial registration:**

NFEC, NFEC-2025-021, approved on January 10, 2025.

**Key Points:**

- Radiation exposure and substantial contrast medium doses during CTPA increased patient risks.
- Virtual monoenergetic imaging at 40 keV derived from dual-energy CT enhanced pulmonary vascular visualization.
- The dual-flow mixture technique with dual-energy CT reduced radiation dose and contrast medium volume.

**Graphical Abstract:**

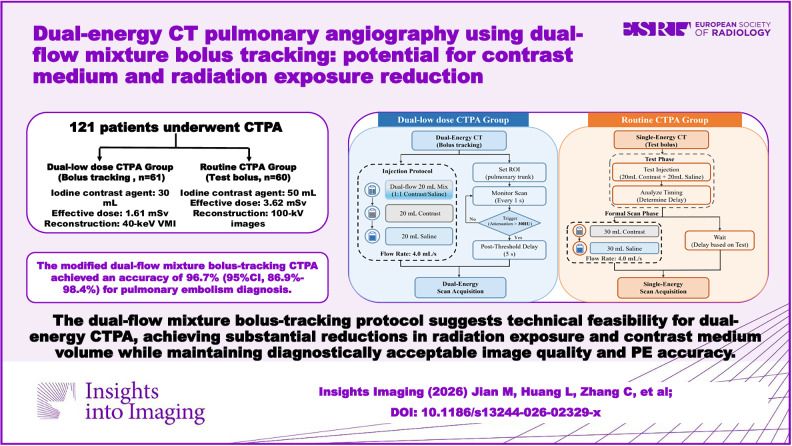

## Introduction

Pulmonary embolism (PE) is the third most common acute cardiovascular disease. With an estimated annual incidence ranging from 39 to 200 cases per 100,000 inhabitants, it is second only to myocardial infarction and stroke [1, 2]. Computed tomography pulmonary angiography (CTPA) has become the first-line imaging modality for suspected PE, owing to its high diagnostic efficiency, widespread availability, and short acquisition time [3–6]. Despite its prevalent clinical use, concerns persist regarding contrast-induced nephropathy. In addition, ionizing exposure during CT scans has been associated with approximately 2% of all newly diagnosed malignancies [7, 8]. Therefore, continuous efforts are focused on enhancing image quality, decreasing radiation dose, and minimizing the use of iodinated contrast agents for diagnosing PE [9–11].

Dual-energy CT (DECT) has demonstrated significant advantages in PE evaluation, particularly by providing additional spectral information and facilitating radiation and contrast dose reduction [9, 10, 12, 13]. Virtual monoenergetic imaging (VMI) reconstructed at specific energy levels (40–190 keV), particularly at lower keV settings, has the potential to enhance image quality in low-contrast conditions [14, 15]. Furthermore, studies have demonstrated that the dose of iodinated contrast medium required for CTPA can be reduced by approximately 50% when utilizing DECT protocols [16, 17].

The traditional test bolus method customizes the peak enhancement triggering time based on the blood flow. However, it necessitates additional contrast agent and extra radiation exposure during the testing phase [18]. Recent studies have reported that the development of mixed fluid bolus tracking CTPA protocols can optimize the contrast agent and radiation doses without compromising image quality. For example, Kristiansen et al. [17] decreased the contrast agent volume to approximately 30 mL using contrast medium dilution while maintaining CTPA diagnostic image quality. However, to the best of our knowledge, the combination of the dual-flow mixture bolus tracking technique and DECT acquisition has not yet been tested in clinical applications.

The aim of the present study was therefore to evaluate the feasibility and image quality of a modified bolus tracking dual low-dose CTPA protocol for low-keV virtual monoenergetic DECT, and to compare it to a standard test bolus single-energy CT protocol.

## Methods

### Patient selection

**Fig. 1:**
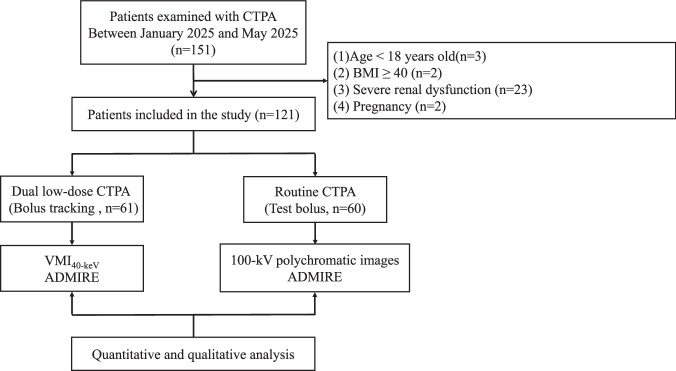
Flowchart of the study population. BMI, body mass index; CTPA, computed tomography pulmonary angiography; VMI, virtual monoenergetic imaging; ADMIRE, advanced modeled iterative reconstruction

This single-center study was approved by our Institutional Review Board (NFEC-2025-021). Written informed consent was obtained from all participants. Patients with suspected PE referred to CTPA were consecutively enrolled in the study between January 2025 and May 2025 (Fig. 1). Exclusion criteria included age of < 18 years, obesity (body mass index (BMI) of ≥ 40 kg/m²), severe renal dysfunction (estimated glomerular filtration rate of < 30 mL/min), or pregnancy.

### Image acquisition

All examinations were performed using a third-generation dual-source CT system (Somatom Force, Siemens Healthcare). Eligible patients with suspected PE were enrolled consecutively and randomly assigned at a 1:1 ratio to the dual low-dose or routine CTPA group using a computer-generated allocation list. Each enrolled patient was assigned to the next available assignment in this sequence before the CTPA scanning. Image acquisition in the dual low-dose CTPA group was triggered using a relative threshold of 30 Hounsfield units (HU), with monitoring performed each second and at a post-threshold delay of 5 s before scan initiation. The contrast injection protocol consisted of an initial 20 mL of a 1:1 dual-flow mixture of iodinated contrast agent and saline, followed by sequential injections of 20 mL of contrast agent and 20 mL of saline, administered at a flow rate of 4.0 mL/s. More detailed information of the scanning and image reconstruction protocols is provided in Table 1 and Supplementary Materials.

**Table 1:** Patient and scan characteristics

Parameter	Dual low-dose CTPA (Bolus tracking, *n* = 61)	Routine CTPA (Test bolus, *n* = 60)	*p*
Patient demographics			
Female/male [*n*]	37:24	31:29	0.319
Age [years]	61.0 (49.0–70.5)	60.0 (43.5–69.00)	0.435
BMI [kg/m^2^]	23.9 (22.0–25.5)	23.9 (21.7–25.4)	0.842
PE [*n*]	13 (21.3%)	19 (31.7%)	0.197
Scanning parameters			
Automatic tube current selection	Yes	Yes	-
Automatic tube voltage control	N/A	N/A	-
Pitch	1.2	1.2	-
Tube current [ref.mAs]	60/46	300	-
Tube potential [kV]	90/sn150	100	-
Rotation time [s]	0.25	0.25	-
Trigger threshold [HU]	30	N/A	-
Injection parameters			
Injection procedure	Dual-flow technique	N/A	-
Contrast medium volume [mL]	10 + 20	20 + 30	-
Normal saline volume [mL]	10 + 20	20 + 30	-
Iodine delivery rate [mg/s]	1400	1400	-
Injection rate [mL/s]	4	4	-
Reconstruction parameters			
Kernel	Qr36	Bv36	-
Slice thickness [mm]	1.5	1.5	-
Iterative reconstruction algorithm	ADMIRE	ADMIRE	-
Strength of iteration	3	3	-

Technical parameters of applied protocols

Numbers are median (interquartile range) or count (percentage)

*ADMIRE* advanced modeled iterative reconstruction (Siemens Healthiness)

### Quantitative image analysis

Quantitative image analysis was performed by a radiologist (M. J.) with three years of experience, who placed circular regions of interest (ROIs) in the main pulmonary artery trunk, left and right pulmonary arteries, right upper segmental artery, left lower segmental artery, and periscapular musculature. The ROIs covered approximately two-thirds of the vessel cross-sectional area to ensure measurement accuracy. Each was measured three times and averaged. Measurements were carried out on 100-kV axial polyenergetic CT images or utilizing VMI at 40, 50, 60, and 70 keV.

The left teres major muscle served as the reference tissue for calculating the contrast-to-noise ratio (CNR) and signal-to-noise ratio (SNR) of the vascular structures because muscle tissue density was relatively uniform and less affected by contrast enhancement. The CNR was calculated using the following formula:

$${{\mathrm{CNR}}}=\frac{{{\mathrm{mean}}}\,{{\mathrm{attenuation}}}\,{{\mathrm{value}}}n\,\left[{{\mathrm{HU}}}\right]-{{\mathrm{attenuation}}}\,{{\mathrm{value}}}\,{{\mathrm{of}}}\,{{\mathrm{teres}}}\,{{\mathrm{major}}}\,{{\mathrm{muscle}}}\,[{{\mathrm{HU}}}]}{{{\mathrm{mean}}}\,{{\mathrm{noise}}}\,{{\mathrm{of}}}\,{{\mathrm{teres}}}\,{{\mathrm{major}}}\,{{\mathrm{muscle}}}\,[{{\mathrm{SD}}}\,{{\mathrm{of}}}\,{{\mathrm{HU}}}]}$$

The SNR was calculated based on the formula:

$${{\mathrm{SNR}}}=\frac{{{\mathrm{mean}}}\,{{\mathrm{attenuation}}}\,{{\mathrm{value}}}\,[{{\mathrm{HU}}}]}{{{\mathrm{mean}}}\,{{\mathrm{noise}}}\,{{\mathrm{of}}}\,{{\mathrm{the}}}\,{{\mathrm{vessel}}}\,[{{\mathrm{SD}}}\,{{\mathrm{of}}}\,{{\mathrm{HU}}}]}$$

A figure of merit (FOM), defined as the ratio of CNR² to the effective dose (ED) for each series, was used to evaluate the relationship between CNR and radiation dose according to the following formula [19]:

$${{{\mathrm{FOM}}}}_{{{\mathrm{CNR}}}}=\frac{{{{\mathrm{CNR}}}}^{2}}{{{\mathrm{effective}}}\,{{\mathrm{dose}}}}$$

### Qualitative image analysis

**Fig. 2:**
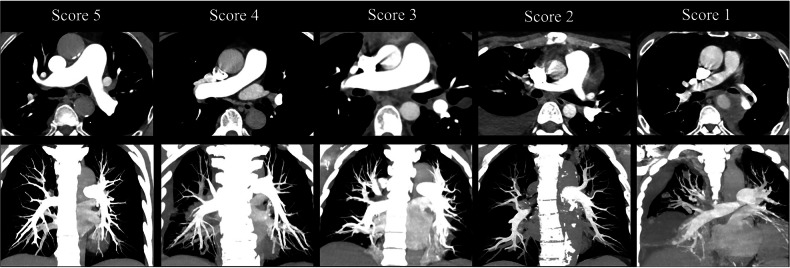
Schematic of the subjective scoring criteria for superior vena cava artifacts (top panel) and visualization of pulmonary arterial branches (bottom panel)

Qualitative image analysis evaluated image noise, superior vena cava artifacts, and pulmonary arterial branch visualization, which was performed on a five-point scale (5, best; 1, worst) by two radiologists (X.Q. and L.H., with 3 and 5 years of cardiovascular imaging experience, respectively) independently. The radiologists were blinded to the image reconstruction techniques and assessed the images in random order. The noise and superior vena cava artifacts were analyzed based on the VMI data for the dual low-dose CTPA group and 100-kV axial polyenergetic CT images for the routine CTPA group. Pulmonary arterial branch visualization was assessed on maximum intensity projection (MIP) images. Detailed subjective rating criteria are provided in Fig. 2 and Supplementary Table S1.

Suboptimal cases were defined as those with a main pulmonary artery attenuation below 211 HU in the dual low-dose CTPA group. VMI reconstructed at low keV values was used to salvage these suboptimal scans.

### PE detection and reference standard

The reference standard was established by an expert committee consisting of three cardiovascular radiologists with 10, 13, and 19 years of experience. They were each required to review imaging reports, diagnose the presence or absence of PE, and record the anatomic location of any clots. Final decisions on the reference standard were made by consensus. The emboli location was defined as either central (main pulmonary artery trunk, left and right pulmonary arteries, and lobar arteries) or peripheral (segmental and subsegmental arteries). Twenty peripheral arteries were evaluated in all patients, including three segmental (subsegmental) arteries in the upper lobe, two in the middle or lingual lobe, and five in the inferior lobe for each lung [20].

For the PE detection reader study, two radiologists (X.Q. and L.H.) independently assessed the image series for the presence or absence of an embolism in a randomized order. A third senior radiologist (C.Y.) with 12 years of experience determined the final result in cases of disagreement.

### Radiation dose estimation

The volume CT dose index (CTDIvol) and dose-length product (DLP) were recorded during the pulmonary arterial enhancement phase and the monitoring or test injection phase. The ED in mSv was then estimated by multiplying the DLP by the standard conversion factor of 0.014 mSv/mGy·cm.

### Study endpoints

Technical feasibility and CTPA protocol image quality comparison served as the primary study endpoints. Secondary endpoints of radiation exposure and contrast medium dosage reduction were compared between the two protocols.

### Statistical analysis

The non-inferiority study was designed to compare the accuracy rates between two groups. The sample size was calculated using a non-inferiority test for two independent proportions (PASS v2025; unpooled *Z*-test). A total of 54 participants per group were required, assuming a reference accuracy of 96.5%, a non-inferiority margin of −10%, one-sided α of 0.025, and 80% power.

Data were analyzed using SPSS software (version 25; IBM) and R (version 4.4.2). Continuous variables with a normal distribution were expressed as the mean ± standard deviation (SD) and or the median (interquartile range). Categorical data were presented as numbers (percentages) and analyzed using the Chi-square or Fisher’s exact test. The independent sample *t*-test was used for normally distributed continuous variables, and the Mann–Whitney *U*-test was used for categorical or non-normally distributed variables. All statistical tests had a two-tailed α level of 0.05. Interobserver agreement was assessed using the intra-class correlation coefficient or Cohen’s kappa analysis. Sensitivity, specificity, positive predictive value, negative predictive value, and accuracy of the two CTPA protocols for the PE detection were calculated, and their 95% confidence intervals were estimated using nonparametric bootstrap resampling methods. The degree of agreement was categorized as poor (< 0.20), fair (0.20–0.40), moderate (0.40–0.60), good (0.60–0.80), or excellent (> 0.80). A *p* value of less than 0.05 was considered statistically significant.

## Results

### Patient population

No statistically significant difference (*p* = 0.197–0.842) was found in the distribution of age, BMI, sex, or PE incidence between the two groups (Table 1). Two suboptimal examinations in the dual low-dose CTPA group were salvaged using VMI.

### Quantitative image analysis

VMI at 40 keV in the dual low-dose CTPA group showed the highest CNR of 29.8 (24.0–44.2) in all pulmonary arteries among the 40–70 keV levels (Supplementary Fig. S1). The dual low-dose CTPA group showed significantly higher attenuation in the main pulmonary trunk and bilateral pulmonary arteries compared to those in the routine CTPA group (all, *p* < 0.001; Table 2). Otherwise, the dual low-dose CTPA images yielded significantly higher noise and lower corresponding SNR compared to those in the routine CTPA group (all, *p* < 0.001). Additionally, no significant difference in CNR was found in the pulmonary trunk and the right and left pulmonary arteries between the two groups (*p* = 0.115–0.322). The FOM_CNR_ values in the dual low-dose CTPA group were significantly higher than those in the routine CTPA group, except for the left lower segmental artery (*p* < 0.05).

**Table 2:** Comparison of quantitative image quality measurements

Parameter	Dual low-dose CTPA (Bolus tracking, *n* = 61)	Routine CTPA (Test bolus, *n* = 60)	*p*
	40-keV VMIs	100-kVp	
Pulmonary trunk			
CT attenuation [HU]	1027.0 ± 287.6	391.8 ± 109.0	< 0.001
Noise [HU]	44.5 (37.2–50.8)	10.0 (7.7–12.3)	< 0.001
SNR	22.9 (18.4–26.5)	39.3 (29.2–49.7)	< 0.001
CNR	33.6 (25.7–49.5)	41.0 (27.5–55.2)	0.115
FOM_CNR_	711.4 (424.0–1480.2)	461.5 (224.8–928.6)	0.006
Left pulmonary artery			
CT attenuation [HU]	968.1 ± 285.9	361.7 ± 90.9	< 0.001
Noise [HU]	48.2 (43.2–54.7)	12.3 (9.3–15.0)	< 0.001
SNR	19.4 (15.5–22.8)	28.3 (23.7–37.7)	< 0.001
CNR	29.9 (24.5–44.2)	35.2 (26.1–50.6)	0.219
FOM_CNR_	544.1 (304.9–1361.6)	362.1 (195.4–706.5)	0.001
Right pulmonary artery			
CT attenuation [HU]	997.4 ± 277.7	366.4 ± 95.4	< 0.001
Noise [HU]	53.3 (44.8–65.6)	14.0 (10.4–18.8)	< 0.001
SNR	17.5 (14.6–22.1)	25.4 (18.1–32.6)	< 0.001
CNR	31.9 (24.3–46.5)	36.6 (25.8–49.7)	0.322
FOM_CNR_	634.7 (344.0–1507.7)	382.9 (165.6–738.8)	0.001
Right upper segmental artery			
CT attenuation [HU]	901.8 ± 254.8	376.1 ± 107.7	< 0.001
Noise [HU]	38.2 (27.9–49.5)	12.0 (9.5–15.4)	< 0.001
SNR	22.9 (17.0–28.7)	30.9 (23.2–39.8)	< 0.001
CNR	29.2 (21.9–41.7)	37.1 (25.4–52.7)	0.018
FOM_CNR_	562.5 (275.2–956.5)	361.9 (187.1–824.5)	0.049
Left lower segmental artery			
CT attenuation [HU]	838.4 ± 263.0	353.1 ± 95.9	< 0.001
Noise [HU]	38.8 (31.0–52.1)	10.5 (8.5–12.5)	< 0.001
SNR	20.5 (15.2–27.3)	32.3 (23.6–41.6)	< 0.001
CNR	26.6 (20.8–39.7)	34.8 (24.2–49.1)	0.013
FOM_CNR_	400.0 (260.7–1054.9)	348.6 (147.6–698.4)	0.063
Muscle			
CT attenuation [HU]	50.7 (41.8–58.8)	59.7 (56.8–62.3)	< 0.001
Noise [HU]	27.3 (22.4–33.4)	8.3 (6.8–9.3)	< 0.001

Variables are presented as mean ± SD when normally distributed or as median

(interquartile range) for non-normal distributions

*VMIs* virtual monoenergetic images, *SNR* signal-to-noise ratio, *CNR* contrast-to-noise ratio, *FOM* figure of merit

### Qualitative image analysis

There was no significant difference in subjective scores between the two groups in terms of pulmonary artery visualization (*p* = 0.660 and 0.763; Table 3). Both radiologists rated 90.2% of patients (55/61) with Score 4 or 5 in the dual low-dose CTPA group compared to 81.7% (49/60) or 80% (48/60) in the routine CTPA group. Both radiologists rated the dual low-dose CTPA group as superior to the routine CTPA group when evaluating the superior vena cava beam-hardening artifacts (both *p* < 0.05). The qualitative image noise was rated as inferior in the dual low-dose CTPA group by two radiologists (both *p* < 0.001), with only five cases (8.2%) scoring 2 or 1.

**Table 3:** Qualitative image quality analysis of pulmonary arteries

	Reader 1			Reader 2		
	Dual low-dose CTPA (Bolus tracking, *n* = 61)	Routine CTPA (Test bolus, *n* = 60)	*p*	Dual low-dose CTPA (Bolus tracking, *n* = 61)	Routine CTPA (Test bolus, *n* = 60)	*p*
	40-keV VMI	100-kVp		40-keV VMI	100-kVp	
Pulmonary arterial branch visualization						
Score 5	34 (55.7%)	33 (55%)	0.660	33 (54.1%)	37 (61.7%)	0.763
Score 4	21 (34.4%)	16 (26.7%)		22 (36.1%)	11 (18.3%)	
Score 3	3 (4.9%)	6 (10%)		2 (3.3%)	7 (11.7%)	
Score 2	1 (1.6%)	4 (6.7%)		2 (3.3%)	4 (6.7%)	
Score 1	2 (3.3%)	1 (1.7%)		2 (3.3%)	1 (1.7%)	
κ	0.713	0.664				
Superior vena cava artifacts						
Score 5	7 (11.5%)	5 (8.3%)	< 0.001	18 (29.5%)	9 (15%)	0.002
Score 4	27 (44.3%)	10 (16.7%)		14 (23%)	7 (11.7%)	
Score 3	9 (18%)	10 (16.7%)		8 (13.1%)	10 (16.7%)	
Score 2	15 (24.6%)	19 (31.7%)		13 (21.3%)	14 (23.3%)	
Score 1	3 (4.9%)	16 (26.7%)		8 (13.1%)	20 (33.3%)	
κ	0.541	0.614				
Noise						
Score 5	3 (4.9%)	36 (60%)	< 0.001	3 (4.9%)	37 (61.7%)	< 0.001
Score 4	27 (44.3%)	24 (40%)		21 (34.4%)	22 (36.7%)	
Score 3	26 (42.6%)	0		32 (52.5%)	1 (1.7%)	
Score 2	4 (6.6%)	0		4 (6.6%)	0	
Score 1	1 (1.6%)	0		1 (1.6%)	0	
κ	0.681	0.793				

For group comparison, the chi-square test was used. Numbers are counted and (percentage) or mean ± SD

*VMIs* virtual monoenergetic images

The interreader agreement for the subjective image analysis was moderate to good for the dual low-dose (κ = 0.541–0.713) and routine (κ = 0.614–0.793) CTPA groups.

### PE detection and reference standard

**Fig. 3:**
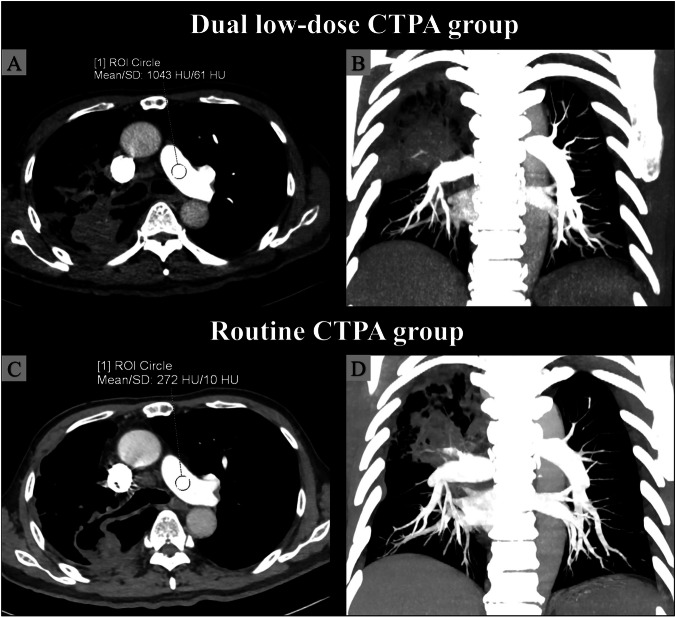
The axial contrast-enhanced CT images and MIP images in a 62-year-old male patient (BMI, 21.6 kg/m²) who underwent CTPA examinations 8 months apart using the VMI at 40-keV derived from dual low-dose CTPA (**A**) and routine CTPA at 100-kV (**C**) protocols. MIP images are generated from the corresponding axial CT data (**B**, **D**). The CT numbers measured in the pulmonary trunk are 1043.0 HU and 272.0 HU, respectively

**Fig. 4:**
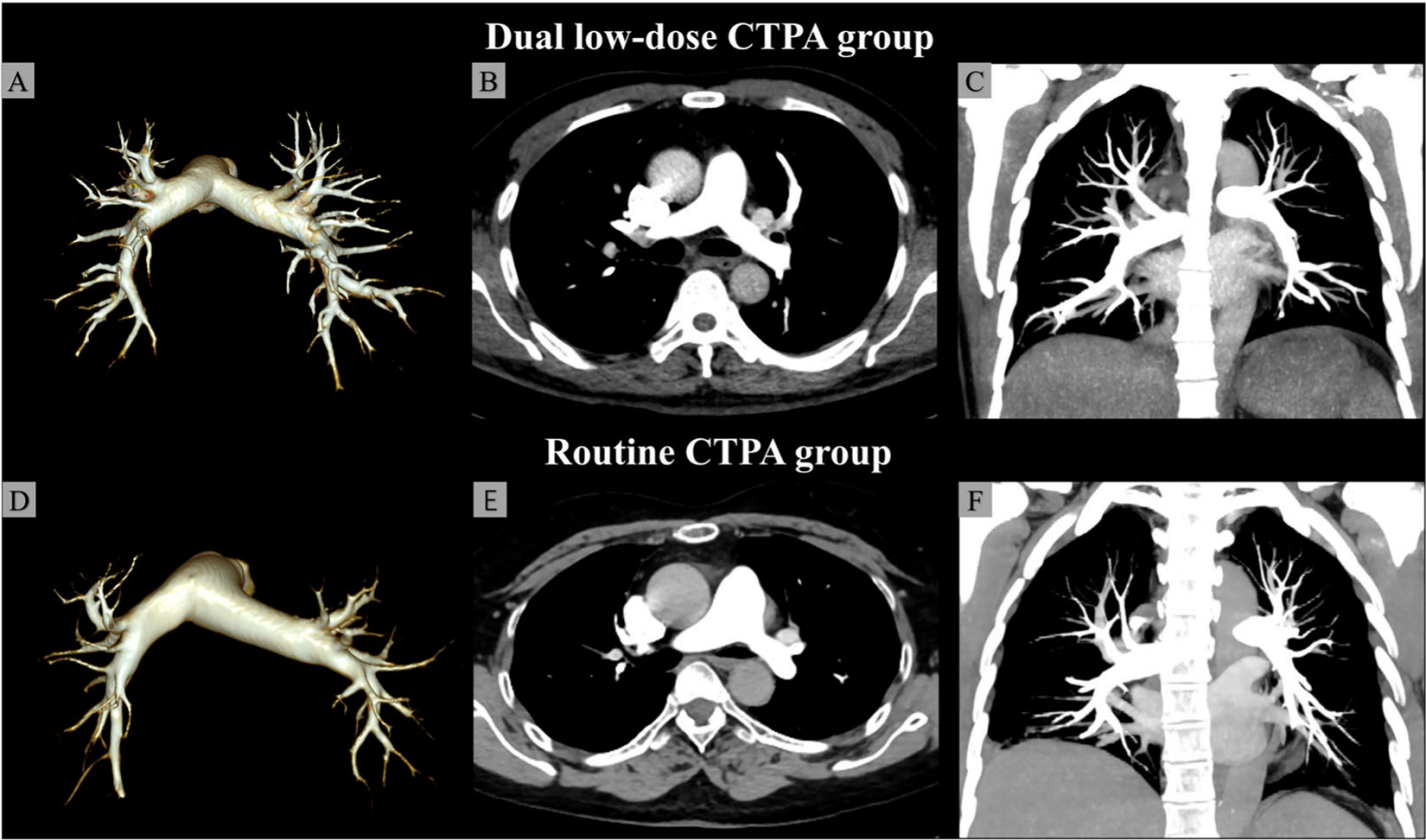
**A**–**C** A 56-year-old male patient with a BMI of 23.7 kg/m^2^, in dual low-dose CTPA, DLP was 105.2 mGy·cm and contrast agent dose was 30 mL; **A** volume rendering (VR), **B**, **C** axial and coronal MIP. **D**, **E** a 53-year-old female patient with BMI 23.97 of kg/m^2^, DLP was 239 mGy·cm, and contrast agent dose was 50 mL; **D** VR, and **E**, **F** axial and coronal MIP

**Fig. 5:**
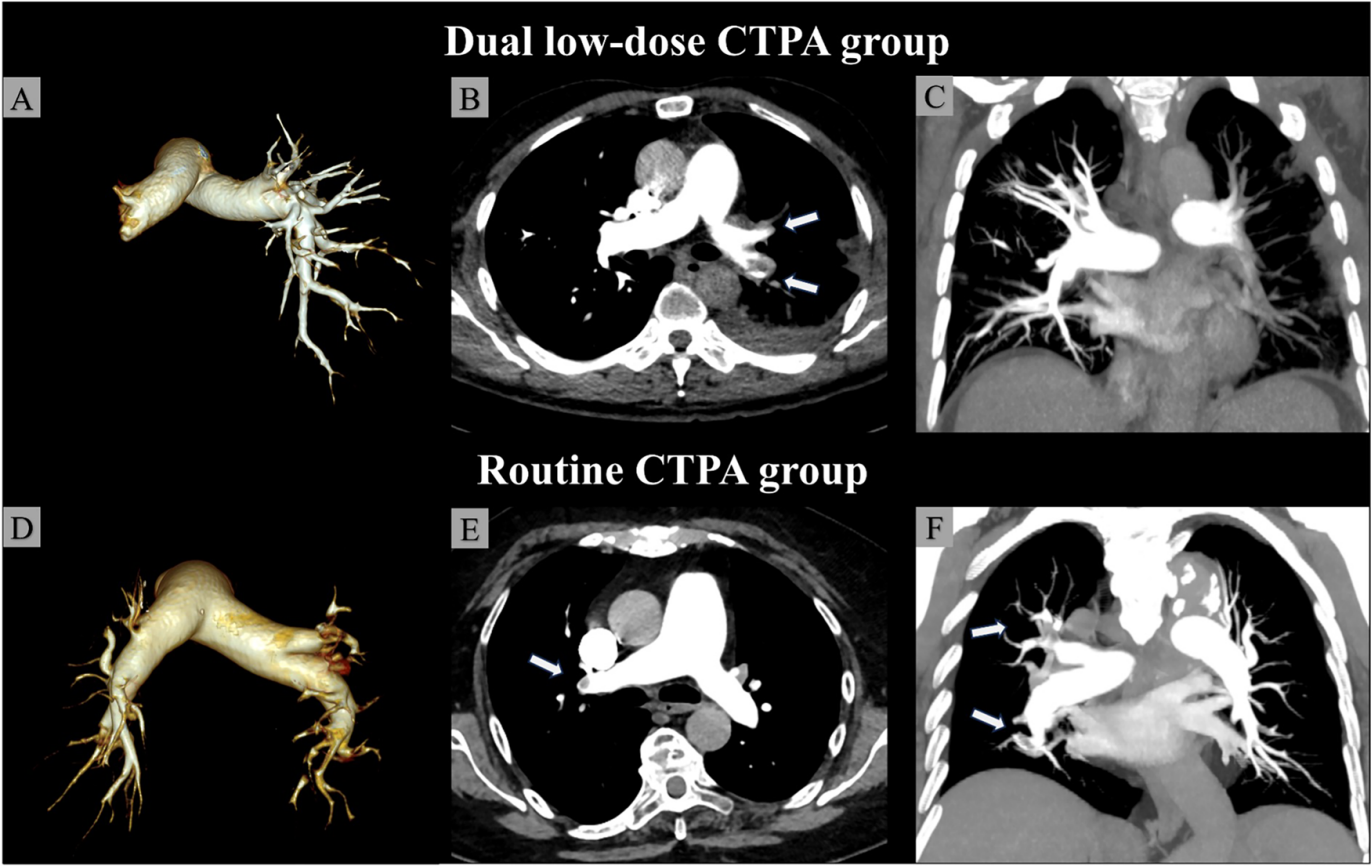
**A**–**C** A 58-year-old male patient with a BMI of 24.4 kg/m^2^ and confirmed PE, in dual low-dose CTPA, DLP was 92.3 mGy·cm and contrast agent dose was 30 mL; **A** VR, **B**, **C** axial and coronal MIP. **D**–**F** A 53-year-old male patient with a BMI of 26.5 kg/m^2^ and confirmed PE, DLP was 314.9 mGy·cm and contrast agent dose was 50 mL; **D** VR, **E**, **F** axial and coronal MIP. White arrows indicate noticeable filling defects on axial imaging and MIP

PE was detected in 13 cases (21.3%) in the dual low-dose CTPA group and 19 cases (31.7%) in the routine CTPA group. The sensitivity, specificity, positive predictive value, negative predictive value, accuracy, and 95% confidence intervals in the two CTPA groups are summarized in Table 4. No significant difference was identified for PE diagnostic accuracy between the two groups (*p* > 0.05). Representative cases are shown in Figs. 3–5.

**Table 4:** Diagnostic accuracy for the PE detection

	Patients		Central PE		Peripheral PE		
	Dual low-dose CTPA (Bolus tracking, *n* = 61)	Routine CTPA (Test bolus, *n* = 60)	Dual low-dose CTPA (Bolus tracking, *n* = 19)	Routine CTPA (Test bolus, *n* = 24)	Dual low-dose CTPA (Bolus tracking, *n* = 30)		Routine CTPA (Test bolus, *n* = 43)
Parameter	40-keV VMI	100-kVp	40-keV VMI	100-kVp	40-keV VMI		100-kVp
Sensitivity	92.3% (95% CI, 63.6%–100%)	94.7% (95% CI, 70.0%–100%)	100% -	100% -	90.0% (95% CI, 71.9%–97.0%)		95.3% (95% CI, 84.2%–100%)
Specificity	97.9% (95% CI, 83.3%–100%)	100% -	100% -	100% -	99.7% (95% CI, 99.2%–99.9%)		99.8% (95% CI, 99.4%–100%)
PPV	92.3% (95% CI, 63.6%–100%)	100% -	100% -	100% -	87.0% (95% CI, 70.4%–96.6%)		95.3% (95% CI, 85.1%–100%)
NPV	97.9% (95% CI, 88.0%–100%)	97.6% (95% CI, 87.8%–100%)	100% -	100% -	99.7% (95% CI, 70.4%–96.6%)		99.8% (95% CI, 99.4%–100%)
Accuracy	96.7% (95% CI, 86.9%–98.4%)	98.3% (95% CI, 91.7%–100%)	100% -	100% -	99.4% (95% CI, 98.7%–99.7%)		99.7% (95% CI, 99.0%–99.8%)
AUC	95.1% (95%CI, 87.3%–100%)	97.4% (95% CI, 92.2%–100%)	-	-	94.8% (95% CI, 89.4%–100%)		97.6% (95% CI, 94.4%–100%)

*CTPA* computed tomography pulmonary angiography, *VMIs* virtual monoenergetic images, *NPV* negative predictive value, *PPV* positive predictive value, *PE* pulmonary embolism

### Radiation dose estimation

The median ED of the dual low-dose CTPA group was significantly lower than that of the routine CTPA group (1.61 mSv vs 3.62 mSv), leading to a 55% radiation dose reduction. Detailed results of the radiation dose parameters are presented in Table 5.

**Table 5:** Estimations of radiation exposure

Parameters	Dual low-dose CTPA	Routine CTPA	*p*
CTDIvol [mGy]	3.06 (2.53–3.73)	7.86 (6.73–8.24)	< 0.001
DLP [mGy·cm]	109.6 (90.4–134.5)	246.2 (227.2–269.5)	< 0.001
DLP^#^ [mGy·cm]	5.70 (4.80–6.50)	12.1 (11.30–12.10)	< 0.001
ED [mSv]^*^	1.61 (1.35–1.97)	3.62 (3.35–3.93)	< 0.001

Radiation dose is shown as median (interquartile range)

*DLP* dose-length product, *CTDI*_vol_ volume CT dose index, *ED* effective dose

^#^Monitoring or testing layer

^*^(DLP + DLP#) × 0.014

## Discussion

Radiation dose and contrast medium volume optimization are major concerns in CTPA. The present study assessed quantitative and qualitative image quality of a dual low-dose DECT CTPA protocol designed to reduce contrast medium volume and radiation dose. The modified dual-flow mixture technique with bolus tracking using 30 mL of contrast medium provided acceptable pulmonary artery branch visualization, while considerably lowering the radiation dose relative to the routine test bolus single-energy CT protocol. Although the dual low-dose approach resulted in approximately 55% lower radiation exposure and a 40% reduction in contrast medium volume, image quality of 40-keV VMI remained adequate for clinical interpretation despite increased noise levels.

Because dual-energy scanning could not be combined with high-pitch mode, the contrast medium volume (30 mL) was higher than that utilized in a prior ultra-high-pitch (pitch = 3.2) study that used 20 mL [5]. The reduction in contrast medium volume also led to decreased beam-hardening artifacts in the superior vena cava, which was consistent with previous findings [21]. Another study used a test bolus approach, which resulted in superior intravascular opacification in CTPA compared to that observed when using the bolus tracking technique [22]. In this work, subjective pulmonary vascular image quality did not differ significantly between the dual low-bolus tracking and routine test-bolus groups. The diagnostic performance analysis in the study was exploratory and not used as a definitive non-inferiority trial. Although the test-bolus method determined pulmonary arterial peak enhancement more accurately, it necessitated an additional 10–20 mL of contrast agent to measure the time to peak enhancement. Conversely, the dual-flow mixture protocol in the present study used diluted contrast agent during the trigger-to-scan delay, thereby further reducing the overall contrast medium dose.

Recent studies have investigated various dose-saving techniques incorporating reduced tube voltage or current to achieve a substantial radiation dose reduction in CTPA [11, 23, 24]. Deep-learning reconstruction can further reduce noise and radiation dose in low-kV CTPA, as shown by Zhang et al [2]. However, the DECT system in the present study did not support deep-learning reconstruction, which limited the potential for further radiation-dose reduction in the protocol. The study used dual-energy mode with a lowered tube current and tin filtration in the high-voltage tube, with a 55% reduction in effective radiation dose (1.61 mSv vs 3.62 mSv), which was significantly lower than that reported by Abdellatif et al (105/81 ref.mA, 90/150 kV, ED of 2.8 mSv) [25]. The present study revealed significantly higher FOM_CNR_ in the dual low-dose group compared to that in the routine group, leveraging the considerable dose savings achieved. Spectral shaping of the X-ray beam using tin filtration in CT facilitated harmful radiation reduction by filtering out low-energy X-ray photons absorbed by body tissues [26]. In addition, the bolus-tracking monitoring scans used to determine the trigger time delivered less radiation than the test-bolus scan when the attenuation threshold for bolus tracking was set to 30 HU (0.08 mSv vs 0.17 mSv).

DECT allows VMI generation at varying monoenergetic levels, enhancing contrast intensity in suboptimal contrast-enhanced studies with promising results [27–29]. Similarly, the present study findings indicated that 40-keV VMI provided the highest CNR, which allowed for the maximal photoabsorption effect of iodine and outperformed 50–70-keV VMI [12, 27, 30]. To reflect real-world reading, 40-keV VMI was intentionally benchmarked from the low-dose DECT protocol against the clinical reference standard 100-kV polyenergetic images. In the dual-low dose protocol, CNR remained comparable while preserving pulmonary artery branch visualization, despite higher noise and lower SNR levels. This apparent discrepancy likely reflected the use of different noise terms for the vascular and the teres major muscle in the SNR and CNR calculations. Recent advances in photon-counting CT and ultra-high-pitch CTPA have shown excellent image quality and dose efficiency. Ultra-high-pitch “flash” modes on dual-source scanners use both X-ray tubes for single-energy acquisition, making high pitch and spectral information mutually exclusive and leaving no option to salvage suboptimal contrast timing [26, 31].

The present study had several limitations. First, it was a single-center study with a relatively limited sample size, particularly for peripheral PE-positive cases. Thus, stratification by BMI or sex was not performed in the subgroup analysis. Second, primary image interpretation was carried out by two junior radiologists, with discrepancies adjudicated by a senior radiologist. This may have limited external validity and generalizability of the findings. Third, the study used a dual-source DECT system from a single device. This vendor-specific implementation of dual-energy acquisition and reconstruction may have limited applicability to platforms from other manufacturers. Therefore, a multicenter validation study across different device platforms in larger cohorts is needed. Finally, although only patients with BMI levels of < 40 kg/m² were included in the investigation, and most participants had preserved renal function, clinical outcomes, such as contrast-induced nephropathy and downstream management, were not assessed in this work. Therefore, applicability to morbidly obese or renally impaired populations needs to be explored in future studies.

## Conclusion

The dual-flow mixture bolus-tracking protocol demonstrated technical feasibility for dual-energy CTPA, achieving substantial reductions in radiation exposure and contrast medium volume while maintaining diagnostically acceptable image quality.

## Supplementary information

ELECTRONIC SUPPLEMENTARY MATERIAL

## Data Availability

The datasets analyzed during the current study are available from the corresponding author upon reasonable request.

## Abbreviations

ADMIRE: Advanced modeled iterative reconstruction
BMI: Body mass index
CNR: Contrast-to-noise ratio
CTDIvol: Volume computed tomography dose index
CTPA: Computed tomography pulmonary angiography
DECT: Dual-energy computed tomography
DLP: Dose-length product
ED: Effective dose
FOM: Figure of merit
HU: Hounsfield units
MIP: Maximum intensity projection
PE: Pulmonary embolism
SD: Standard deviation
SNR: Signal-to-noise ratio
VMI: Virtual monoenergetic imaging
VR: Volume rendering
