# Supplementary material for: Dual-energy CT pulmonary angiography using dual-flow mixture bolus tracking: potential for contrast medium and radiation exposure reduction
Source: Insights Imaging. 2026 Jun 20;17:164. doi: 10.1186/s13244-026-02329-x (PMC13282423; doi:10.1186/s13244-026-02329-x)
Supplement: Supplementary file 1 — ELECTRONIC SUPPLEMENTARY MATERIAL [file 13244_2026_2329_MOESM1_ESM.pdf]

Dual-Energy CT Pulmonary Angiography Using Dual-Flow Mixture Bolus Tracking: Potential for Contrast Medium Volume and Radiation Exposure Reduction

ELECTRONIC SUPPLEMENTARY MATERIAL

Methods

Image Acquisition

All examinations were performed using a third-generation dual-source CT system (Somatom Force, Siemens Healthcare). In the dual-low dose CTPA group, a dual-energy spiral CT scanning protocol was employed with tube voltages of 90/sn150 kV and reference tube currents of 60/46 mAs. The region of interest (ROI) was positioned in the main pulmonary artery at the level of the tracheal bifurcation. Image acquisition was triggered using a relative threshold of 30 Hounsfield units (HU), with monitoring performed each second and a post-threshold delay of 5 seconds before scan initiation. The contrast injection protocol consisted of an initial 20 mL of a 1:1 dual-flow mixture of iodinated contrast agent and saline, followed by sequential injections of 20 mL contrast agent and 20 mL saline, administered at a flow rate of 4.0 mL/s.

In the routine CTPA group, a single-energy spiral scanning protocol was utilized with a tube voltage of 100 kV and a reference tube current of 300 mAs. The contrast injection protocol comprised an initial test injection of 20 mL of iodinated contrast agent mixed with 20 mL of saline, followed by a formal injection of 30 mL contrast agent and 30 mL saline at a flow rate of 4.0 mL/s.

Iodinated contrast medium was applied (Ioversol®, 350 mgI/mL; Jiangsu Hengrui Pharmaceuticals) via antecubital vein using an automated injector.

Image Reconstruction

Images were reconstructed using the Advanced Modeled Iterative Reconstruction algorithm (ADMIRE, Siemens Healthcare) with the Qr36 or BV36 standard convolution kernel at a reconstruction strength of 3. For dual-energy linear blending images, a blending ratio of α = 40% (40% from the high-voltage tube and 60% from the low-voltage tube) was used. Maximum intensity projection (MIP) images were reconstructed with a thickness of 15 mm and an increment of 5 mm. Multiplanar reconstruction images were reconstructed with thickness of 1.5 mm and an increment of 0.8 mm, using the VMI at 40 keV and 100 kV axial polyenergetic CT images.

Supplementary Table S1 Qualitative image quality evaluation criteria

Likert scale rating	Definition
Noise	
5	Noise is almost imperceptible.
4	Mild image noise is present.
3	Image noise is present, but pulmonary arterial evaluation is not affected.
2	Image noise is present, with noticeable impact on pulmonary arterial diagnosis.
1	Severe noise, image quality is inadequate for diagnostic purposes or exclusion of pulmonary embolism.
Superior vena cava artifacts	
5	No linear beam-hardening artifacts are observed in the superior vena cava.
4	Few beam-hardening artifacts are seen in the superior vena cava, but they do not affect the pulmonary artery wall.
3	Beam-hardening artifacts in the superior vena cava are apparent, with minor impact on the pulmonary artery wall.
2	Prominent beam-hardening artifacts in the superior vena cava, with a clear effect on the pulmonary artery wall.
1	Severe artifacts are present.
Pulmonary arterial branches visualization	
5	More than 50% of subsegmental branches are clearly visualized, with unambiguous differentiation between pulmonary arteries and veins.
4	More than 50% of subsegmental branches are clearly visualized, and pulmonary veins can be accurately distinguished.
3	More than 50% of subsegmental branches are visible, with acceptable differentiation of pulmonary veins.
2	Less than 50% of subsegmental branches are visible, but pulmonary veins can still be distinguished.
1	Less than 50% of subsegmental branches are poorly visualized, and differentiation of pulmonary veins is difficult

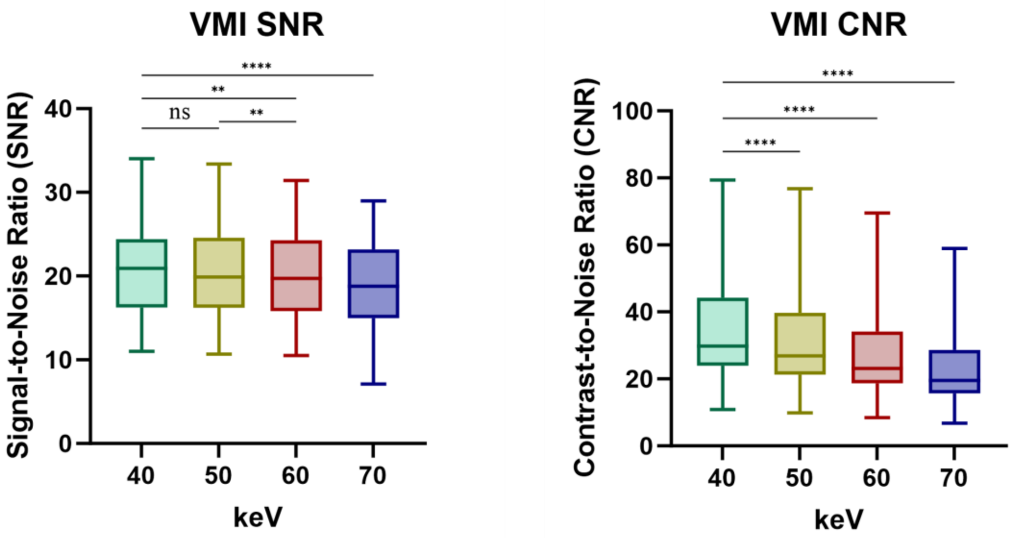

Supplementary Figure S1 Comparison of SNR and CNR in VMIs at 40-70 keV.
